# Case report: Surgical management of ileal duplication cyst in a cat: a case report and review of the literature

**DOI:** 10.3389/fvets.2023.1323088

**Published:** 2023-12-19

**Authors:** Kihoon Kim, Jaehwan Kim, Hwiyool Kim

**Affiliations:** ^1^Department of Veterinary Surgery, College of Veterinary Medicine, Konkuk University, Seoul, Republic of Korea; ^2^Department of Veterinary Medical Imaging, College of Veterinary Medicine, Konkuk University, Seoul, Republic of Korea

**Keywords:** cat, enteric duplication cyst, ileocolic junction, ileum, vomiting

## Abstract

A 6-year-old castrated, mixed breed cat presented with vomiting, anorexia, and lethargy. Ultrasonography and computed tomography revealed a round, well-marginated structure closely associated with the ileum proximal to the ileocolic junction. Exploratory laparotomy revealed a mass originating from the distal end of the ileum, close to the ileocolic junction. The mass did not interact with the intestinal lumen. Excisional biopsy with omentalization was performed without small intestinal resection to preserve the ileocolic junctions. Histopathological examination confirmed the presence of an enteric duplication cyst. The cat recovered uneventfully from surgery and remained asymptomatic postoperatively. No recurrence was identified 4 months after surgery. Enteric duplication cysts are uncommon congenital anomalies that originate in the gastrointestinal tract. They could either be communicating or non-communicating with the intestinal lumen. Enteric duplication cysts can be symptomatic or asymptomatic. Enteric duplication cysts associated with the esophagus, duodenum, and jejunum have also been reported in cats. However, to the best of our knowledge, this is the first reported case of an enteric duplication cyst in the feline ileum. Thus, enteric duplication should be considered a differential diagnosis in cystic masses of the ileum.

## 1 Introduction

Enteric duplication cysts are uncommon congenital anomalies (1) that can occur at any level of the alimentary tract, from the tongue to the anus (2–4). They are lined with smooth muscle and mucosal layers, similar to other parts of the alimentary tract (3, 5, 6). According to the human literature, their occurrence is rare but are considered as differential diagnoses for upper intestinal obstruction, especially in infants (2, 7–9). Clinical signs include partial or complete gastrointestinal tract obstruction, melena, or pancreatitis. However, they can also be incidentally identified (2). Several theories have been proposed to explain the mechanism of intestinal duplications in association with urogenital anomalies or concurrent vertebral malformations (9–11). However, the precise etiology of enteric duplication cysts is unclear (12). To the best of our knowledge, this is the first case report describing an intestinal duplication in the ileum of a cat.

## 2 Case description

A 6-year-old castrated male mixed cat weighing 5 kg presented with vomiting, anorexia, and lethargy. On physical examination, the cat was mildly depressed and slightly dehydrated (5%−8%). The complete blood count and blood electrolyte levels were within the normal range. Serum biochemical panel revealed mild hyperglobulinemia (5.3 mg/dl; reference range [RR: 2.8–5.1]) and hypercholesterolemia (274 mg/dl; RR: 65–225). The immunoreactivity of feline pancreatic lipase was unremarkable. Thoracic radiographic examination revealed no abnormalities. Ultrasonography revealed an exophytic, hypoechoic mass adjacent to the ileum.

**Figure 1:**
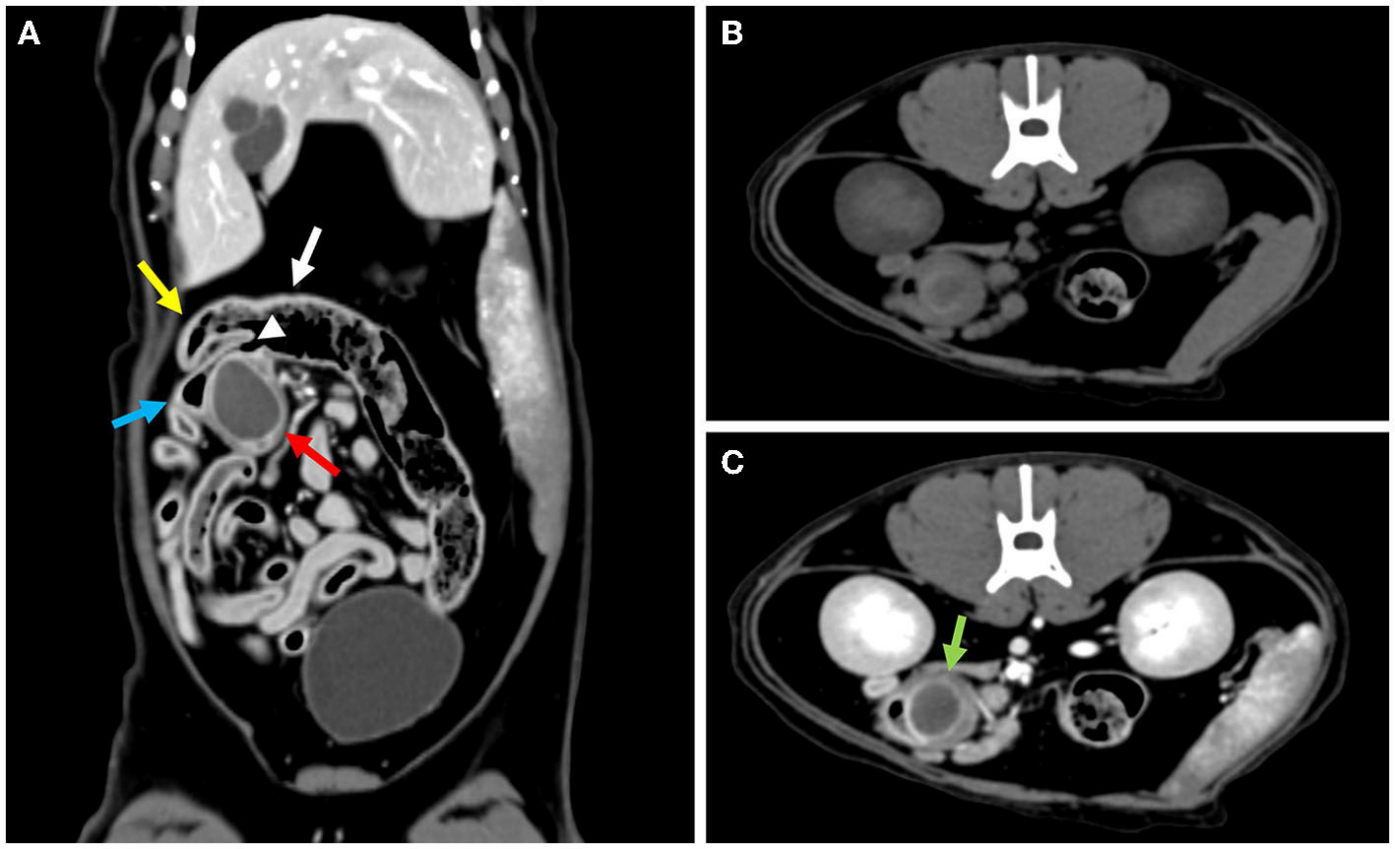
**(A)** The mass (red arrow) adjacent to the ileocolic junction (arrowhead) identified at the distal end of the ileum (blue arrow) on the post-contrast CT image. The cecum (yellow arrow) and colon (white arrow) are not involved in the mass. **(B)** Pre-contrast transverse CT image showing the mass. **(C)** Compared to the pre-contrast transverse CT image, the wall of the mass is enhanced in the post-contrast study (green arrow).

Computed tomography (CT) revealed a round, well-marginated fluid-attenuating protruding mass measuring 19.4 mm × 26.85 mm × 20.55 mm with a thin contrast-enhancing rim at the distal end of the ileum close to the ileocolic junction (ICJ) (Figure 1). The ultrasonographic and CT findings suggested enteric duplication, epidermoid cysts, and abscesses as differential diagnoses.

The patient was premedicated for general anesthesia using intravenous butorphanol (Myungmoon Pharmaceutical, Seoul, Korea) 0.5 mg/kg and ampicillin-sulbactam (Whanin Pharmaceutical, Seoul, Korea) 20 mg/kg. Induction was performed after intravenous injection of 4 mg/kg of alfaxalone (Jurox, Rutherford, Australia). Anesthesia was maintained with isoflurane (Choongwae Pharmaceutical, Seoul, Korea). The patient was administered normal saline (0.9%; Choongwae Pharmaceutical, Seoul, Korea) at a constant infusion rate of 5 mL/kg/h throughout the surgery.

**Figure 2:**
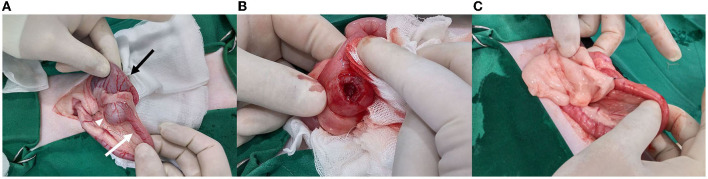
**(A)** A round, firm mass (arrowhead) identified at the distal end of the ileum (white arrow) very close to the ileocolic junction. The mass is located at the mesenteric border of the distal ileum. The colon (black arrow) is grossly normal. **(B)** A hole was made to drain the contents of the mass and to flush the inside of the mass. Further removal of the mass was performed. **(C)** Omentalization performed to secure the omentum to the lesion by simple interrupted sutures.

An exploratory laparotomy revealed a cystic mass originating from the distal end of the ileum, close to the ICJ (Figure 2A). The mass was located at the mesenteric border of the distal ileum. Aspiration of the contents within the mass was attempted but failed because of the contents' viscosity. The contents of the mass were removed through a small hole which was created in the mass (Figure 2B). Subsequently, the remaining parietal cystic wall was removed subtotally. The cystic wall close to the mesenteric border remained to preserve the blood supply to the adjacent ileum. There was no communication between the mass and the adjacent intestinal lumen. The defect was omentalized by securing the omentum to the serosa using simple interrupted sutures (Figure 2C).

**Figure 3:**
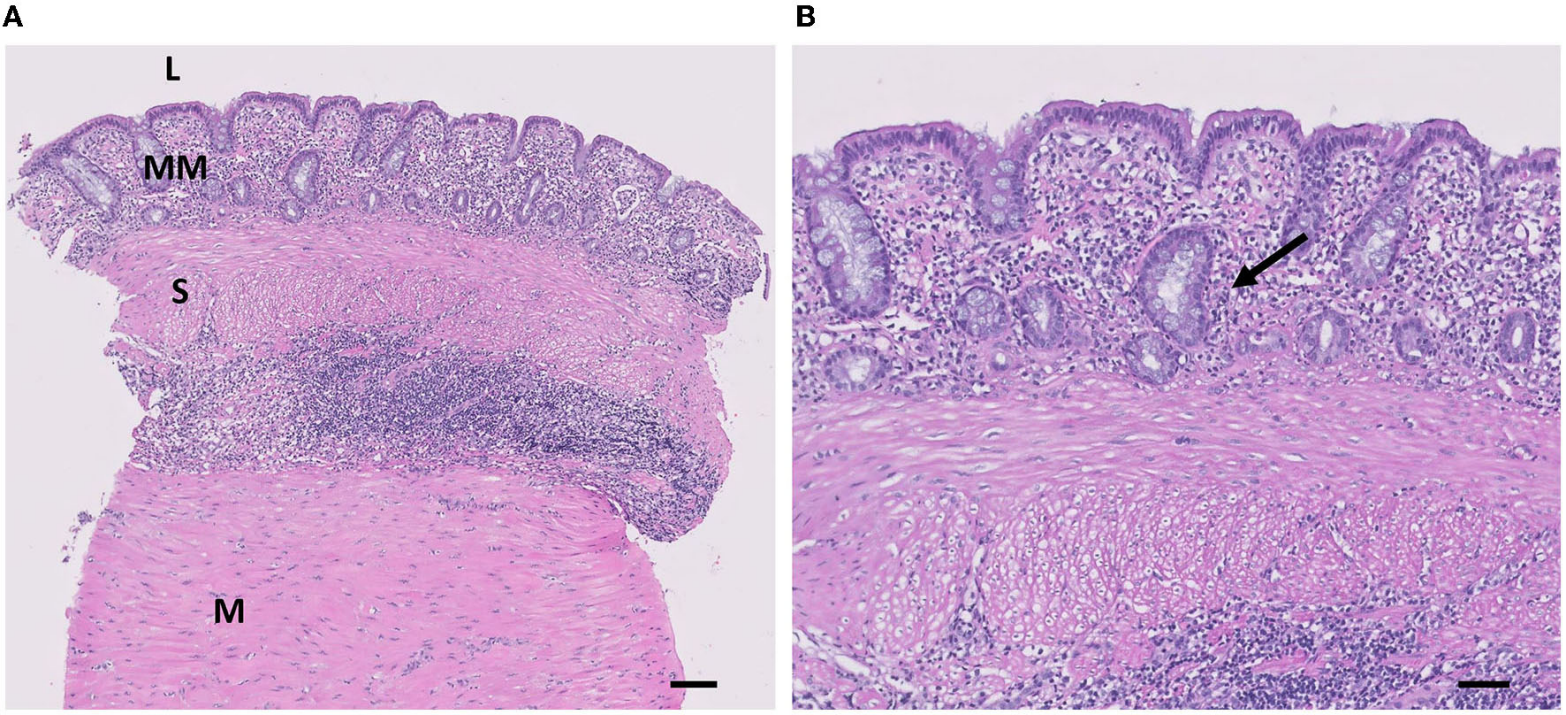
Histological appearance of the lesion. **(A)** The mass is composed of MM, S, and M layers (H&E, scale bar = 100 μm). L, lumen; MM, mucous membrane; S, submucosa; M, Muscularis. **(B)** Mucosal lining of the mass is consisting of columnar epithelial cells and well-developed mucous glands of the inner layer of the wall are shown (arrow) (H&E, scale bar = 50 μm).

The abdominal wall, subcutaneous tissues, and skin were closed routinely. Recovery from anesthesia was uneventful. Cytology of the contents was performed but was nondiagnostic. There was no growth of microorganisms. The cat was discharged on postoperative day 3. Histopathological examination of the cystic mass revealed that the mass had smooth muscles in its walls and was lined by a mucous membrane (Figure 3). The mass had typical layers of intestine consisting of normal mucosa, submucosa, and muscularis (Figure 3A). The mass also revealed intensely mixed, predominantly chronic, mucosal and mural lymphocytic plasmacytic inflammation, with a modest component of active eosinophilic and neutrophilic inflammation. Based on histopathologic findings, the mass was diagnosed as an enteric duplication cyst. Four months after surgery, the referring veterinarian reported no recurrence of clinical signs.

## 3 Discussion

Congenital intestinal abnormalities include diverticula and duplications (2). Considering that the intestinal diverticulum is usually located on the antimesenteric border of the intestine, communicating with the intestinal lumen, as in the case reported here, the diverticulum was excluded from the differential diagnosis (13, 14). Enteric duplication cysts must be located in or close to the gastrointestinal tract and include both a smooth muscle layer and alimentary epithelium (8). All anatomical components were present in this case. Therefore, the final diagnosis of the mass was enteric duplication cyst.

Enteric duplication cysts can occur at any level in the alimentary tract. They can occur at either the antimesenteric or mesenteric border of the small intestine; however, they are generally found on the mesenteric side in humans (13). Eight previously reported cases have described enteric duplication cysts associated with the small intestine in cats (Table 1). Of the eight cases, five cases of enteric duplication cysts occurred in the duodenum (2, 3, 5, 15, 16), two occurred in the jejunum (3, 15), and one occurred in the esophagus and duodenum (16). To our best knowledge, this is the first reported case of an enteric duplication cyst in the ileum.

**Table 1:** Total case of small intestinal duplication cyst in cats.

**Reference**	**Breed**	**Age**	**Site (location)**	**Surgical approach**
Radlinsky et al. (15)	Domestic shorthair	1 year	Jejunum (mesenteric)	Complete resection
Parry-Smith et al. (2)	Unknown	12 weeks	Duodenum (antimesenteric)	Subtotal resection
Kershaw et al. (5)	European shorthair	7 years	Jejunum (mesenteric or antimesenteric)	Complete resection
Bernardé et al. (16)	Domestic shorthair	6 months	Esophagus, duodenum (antimesenteric)	Subtotal resection
Hobbs et al. (17)	Domestic shorthair	15 years	Duodenum (Unknown)	Complete resection
Agut et al. (18)	Siamese	7 months	Duodenum (antimesenteric)	Subtotal resection
Tryon et al. (3)	Domestic shorthair	1.5 years	Jejunum (mesenteric)	Excisional biopsy
Phipps et al. (19)	Domestic shorthair	11 years	Duodenum (antimesenteric)	En bloc resection

Several theories have been proposed to explain the congenital development of enteric duplication cysts (6). According to the human literature, Steiner et al. hypothesized that *in utero* mechanical traction or some vascular events at the proximal side of the diverticulum result in its detachment from the intestine (20). Regarding vascular events, Favara et al. suggested that intrauterine ischemic infarction is the cause of intestinal duplication (21). However, according to the veterinary literature, the exact etiology of an enteric duplication cyst has yet to be established (12).

The clinical signs may differ depending on the location and size of the enteric duplication cyst (3, 19). Of the eight previously reported cases, five were symptomatic, and the most common clinical sign was vomiting (3, 5, 16–18). These are assumed to result from a partial or complete intestinal obstruction (22). The remaining patients were asymptomatic, and enteric duplication cysts were found incidentally (2, 15, 19). In the case reported here, the cat experienced vomiting due to partial obstruction of the ileum, which resolved after surgical management of the mass.

Since abdominal radiography may only reveal a non-specific soft-tissue mass within the abdomen (8), ultrasonography is the most commonly utilized imaging modality for the diagnosis of small intestinal duplications cysts (21). Specifically, if a mass has typical features of the alimentary tract, ultrasonographic examination can be helpful for a definitive diagnosis (23, 24). CT examination may also help diagnose the cystic nature of enteric duplication cysts (4). According to the human literature, enteric duplication cysts may be identified on CT as smoothly rounded, fluid-attenuating structures with thin and slightly contrast-enhanced walls in or adjacent to the intestine.

According to the veterinary literature, the location of the mass can affect the extent of surgical excision of the mass. In 5/8 previously reported cases of enteric duplication cysts associated with the small intestine in feline patients, surgical management involved subtotal resection with preservation of the adjacent small intestine (2, 3, 16, 19, 21). Considering that the mass was located in the duodenum in all five cases, it is assumed that subtotal resection of the mass was performed to preserve the bile and pancreatic ducts. Among the three cases wherein complete resection was performed, the mass in 2/3 cases was located in the jejunum (5, 15). However, in the remaining case, although the mass was located in the duodenum, complete mass resection was performed without complications, likely because the mass was located 3 cm caudal to the duodenal papilla (20). In this case report, subtotal resection was chosen rather than complete removal of the mass because the mass was adjacent to the ICJ. The ICJ regulates intestinal transit, allowing the intestinal contents to move intermittently from the ileum into the colon to ease nutrient absorption. The ICJ also prevents retrograde reflux from the colon into the ileum due to its sphincter properties and the synchronized motility between the ICJ and adjacent intestinal segments (25). Thus, removal of the ICJ leads to a loss of retrograde reflux regulation, resulting in nutrient malabsorption and inflammation of the ileum due to colonic bacteria (26). Severe diarrhea, weight loss, and tenesmus lasting up to 6 months after surgery have been reported after ICJ removal for megacolon management (27, 28). In the case reported here, since the mass was located very close to the ICJ, damage to the ICJ was inevitable in removing the mass completely. Based on the ultrasonographic and CT findings, the mass was assumed to be benign, and debulking surgery was performed rather than complete excision of the mass, preserving the ICJ.

The prognosis of enteric duplication cysts associated with the small intestine is good if there is no recurrence or malignant transformation of the cyst (16, 17). Previously, Bernardé et al. reported multiple enteric duplications originating in the caudal esophagus and duodenum. Recurrence of the esophageal cyst with herniation of the spleen and left lateral lobe of the liver into the thoracic cavity was identified 1 year after repetitive surgical excision at the time of initial surgery. The spleen and the hepatic lobe were restored into the abdomen and the cyst was extensively removed. However, despite additional revision surgery, the cat did not recover from the anesthesia and died postoperatively (16). Hobbs et al. also reported the recurrence of a duodenal duplication cyst that was diagnosed as a duplication cyst with malignant transformation at initial surgery (17).

## 4 Conclusion

In conclusion, in our case, based on the histological examination, the final diagnosis was intestinal duplication. To the best of our knowledge, this is the first reported case of intestinal duplication in the ileum of a cat, along with CT findings. Thus, enteric duplication should be considered a differential diagnosis in cases of ileal masses.

## Data availability statement

The original contributions presented in the study are included in the article/supplementary material, further inquiries can be directed to the corresponding author.

## Ethics statement

Written informed consent was obtained from the participant/patient(s) for the publication of this case report.

## Author contributions

KK: Conceptualization, Investigation, Methodology, Writing – original draft, Writing – review & editing. JK: Methodology, Writing – review & editing. HK: Conceptualization, Validation, Writing – review & editing.
